# A Meta-Analysis of the Acceptance of Artificial Intelligence in Psychotherapy

**DOI:** 10.3390/bs16091552

**Published:** 2026-09-02

**Authors:** Meng Fan, Xiaojing Xie, Tiansheng Xia

**Affiliations:** 1 Mental Health Education Center, Guangdong University of Technology, Guangzhou 510006, China; 2 School of Art & Design, Guangdong University of Technology, Guangzhou 510090, China

**Keywords:** artificial intelligence, psychotherapy, acceptance, heterogeneity, meta-analysis

## Abstract

In recent years, artificial intelligence (AI) technology has demonstrated potential in psychotherapy. However, there is currently a lack of systematic research that comprehensively assessed public acceptance of AI-based psychotherapy. The purpose of this study is to use meta-analysis to explore public acceptance of AI in psychotherapy and the sources of heterogeneity. A random-effects model was employed, using Hedges’ g as the effect size, to pool the differences in acceptance between the AI group and the conventional psychotherapy group. Subgroup analysis and meta-regression were used to explore the sources of heterogeneity. The review included a total of 10 studies involving 1967 participants. Meta-analysis revealed no statistically significant overall difference in acceptance between AI-based psychotherapy and conventional therapy; however, substantial heterogeneity was observed across studies, indicating that this pooled estimate should be interpreted cautiously. Subgroup analysis suggested that the type of conventional psychotherapy, cultural background, and AI type may contribute to variations in acceptance; however, these findings should be interpreted cautiously due to the limited number of studies in some subgroups. Meta-regression analysis indicated that studies with later publication dates and larger sample sizes reported relatively lower AI acceptance. Public acceptance of AI-based psychotherapy may be influenced by multiple factors, including AI type, cultural background, and characteristics of comparison conditions. Future research should focus on cultural differences, the professionalism of AI tools, and the quality of research methodology to more clearly define the appropriate contexts for AI in psychotherapy.

## 1. Introduction

The concept of artificial intelligence was first proposed by John McCarthy in 1955 (McCarthy et al., 2006). Over the past few decades, artificial intelligence has continued to evolve; after 70 years of development, it has appeared in various industries and transformed people’s perceptions. Particularly since the 21st century, its development has been rapid, and it has been widely applied in multiple fields and plays a crucial role, significantly altering every aspect of our lives and ushering in the “Age of Artificial Intelligence” (Thakkar et al., 2024). Against this backdrop, and given the reality that over 970 million people worldwide suffer from mental illness and many lack access to healthcare services (GBD 2019 Mental Disorders Collaborators, 2022), AI—as an innovative tool—is having a significant impact on the field of mental health through prevention and treatment. AI tools can identify high-risk populations to prevent the development of more severe mental illnesses, thereby accelerating the intervention process (Ettman & Galea, 2023). Despite continuous technological advancements, public and patient acceptance of AI in psychotherapy remains a key factor influencing its adoption. Acceptance not only affects the practical effectiveness of the technology but also directly impacts treatment adherence and efficacy. Public concerns regarding data security and the empathy capabilities of AI-based psychotherapy tools are among the reasons for their reluctance to accept AI-based treatments (Aktan et al., 2022).

In this meta-analysis, acceptance of AI-based psychotherapy is conceptualized as a broader construct reflecting individuals’ overall attitudes, evaluations, and willingness to engage with AI-based psychotherapy. Consistent with the Technology Acceptance Model (TAM) and related technology acceptance theories (Davis, 1989), acceptance is regarded as a multidimensional process involving individuals’ cognitive evaluations, attitudes, and behavioral intentions toward adopting a technology. Therefore, acceptance may be operationalized through different indicators, including behavioral intention, general attitudes, perceived acceptability, satisfaction, and trust-related evaluations. Although these indicators represent different aspects of user experience, they share a common focus on individuals’ psychological readiness and overall evaluation of adopting AI-based psychotherapy.

Some researchers have already investigated the relationship between the potential applications of AI in psychotherapy and its actual acceptance, but the findings are inconsistent. Some studies have found that only a very small number of participants expressed a willingness to seek mental health support from chatbots, and participants’ attitudes toward chatbots providing psychological support were significantly less positive than their attitudes toward conventional human-provided mental health services (Rackoff et al., 2025). Conversely, other studies have demonstrated that AI can be considered equivalent to human therapists (Kuhail et al., 2024). Since AI-related research is relatively new, its application in the field of psychotherapy remains a cutting-edge area of exploration. At the same time, because psychotherapy is highly influenced by individual factors, evaluations of AI-assisted psychotherapy vary widely, often resulting in divergent opinions or even two extreme attitudes. To date, no study has systematically synthesized and quantified the evidence regarding public acceptance of AI in psychotherapy through meta-analysis. Therefore, the application of meta-analysis methods is essential for research on the use of AI in psychotherapy.

Although most existing studies have small sample sizes, as artificial intelligence technology continues to advance and public awareness of mental health grows, research on the application of artificial intelligence in psychotherapy will become a major topic of discussion. Therefore, this study conducted a meta-analysis of 10 studies published in recent years to comprehensively evaluate the acceptance of AI in psychotherapy compared to conventional psychotherapy methods between 2017 and 2026, explore key factors influencing acceptance, analyze heterogeneity among existing studies and its sources, and provide evidence-based support for the future promotion and application of AI in psychotherapy.

The theme of this study holds significant theoretical and practical value. It systematically integrates existing evidence to reveal the overall effect of acceptance and its moderating factors. In practice, the research results can provide references for psychological service providers, technology developers, and policy makers, making the design of AI for psychological therapy more reasonable and facilitating its effective promotion. In the remainder of this article, the research methods, results, discussion, and conclusion will be elaborated in sequence, and the report will follow the PRISMA guidelines.

## 2. Methods

This study was conducted in accordance with the 2020 PRISMA (Preferred Reporting Items for Systematic Reviews and Meta-Analyses) guidelines. The PRISMA checklist has been completed and is included in the Supplementary Materials. This review was not registered in a public registry (e.g., PROSPERO) due to the funding deadlines associated with the research project, which limited the opportunity for formal registration during the initial stage of the review. Despite the absence of prior registration, the review process followed PRISMA 2020 recommendations, including predefined eligibility criteria, systematic literature searching, and transparent study selection procedures.

### 2.1. Search Strategy

This study searched the Web of Science, PubMed, Google Scholar, CNKI, and Wanfang databases for literature related to the acceptance of AI applications in psychotherapy. The search scope included academic papers published up to January 2026. Taking Web of Science as an example, Boolean operators (AND, OR, and NOT) were used with the following search string: (“AI” OR “artificial intelligence” OR “chatbot” OR “conversational agent”) AND (“mental health” OR “psychotherapy” OR “counseling”) AND (“attitude” OR “acceptance” OR “acceptability” OR “satisfaction”).

### 2.2. Inclusion Criteria

Studies comparing the acceptance of artificial intelligence in psychotherapy with that of conventional psychotherapy methods, as reported in the literature, were eligible for inclusion in this study. The inclusion criteria for articles were: (1) acceptance-related outcomes toward AI-based psychotherapy measured using Likert-type scales were eligible for inclusion, including willingness/intention to use, attitudes, perceived acceptability, satisfaction, trust-related evaluations, and other indicators reflecting users’ overall evaluation or readiness to engage with AI-based psychotherapy; (2) studied conversational AI models, such as ChatGPT and Woebot; and (3) conventional human-based treatment methods used as controls, such as counseling by human therapists and bibliotherapy. The exclusion criteria for articles were: (1) no report on the acceptance of conventional psychological treatment; (2) missing data; (3) theoretical studies and review articles.

### 2.3. Data Extraction and Risk of Bias Assessment

Two researchers independently conducted the literature screening and data extraction. The information extracted included the first author, year of publication, study location, sample size, characteristics of the study participants, and the methods and data for the experimental and control groups. After completion, the data were cross-checked; any discrepancies were resolved through consultation with a third researcher.

No additional data were requested from study authors, as all necessary information was available from the published reports. For studies that did not directly report standard deviations, we derived them by extracting other relevant values from the reports (e.g., through conversion using standard errors, confidence intervals, or *t*-values). The original and imputed standard deviations are reported in Table 1 to enhance transparency and facilitate verification of effect size calculations. For example, among the included studies, Fitzpatrick et al. (2017) reported means and *t*-test results (*t*_48_ = 3.99, *p* < 0.001) when comparing satisfaction levels, but did not provide standard deviations. We derived the pooled standard deviation using standard formulas based on the *t*-value and its degrees of freedom.

Based on the criteria proposed by Kmet et al. (2004), this comprehensive assessment tool comprises 14 items designed to evaluate quantitative studies, including study design, sample selection, sample characteristics, measurement instruments, statistical methods, presentation of results, and consideration of confounding factors. Each criterion is scored on a 4-point scale (Yes = 2, Partially Yes = 1, No = 0, Not Applicable = NA). The detailed risk of bias assessment results for each study and each domain are provided in Supplementary File S3. Overall judgments indicated that all included studies were classified as having a low risk of bias. Because no study was identified as having a high risk of bias, a risk-of-bias-based sensitivity analysis was not performed. Instead, a leave-one-out sensitivity analysis was conducted to evaluate the robustness of the pooled effect estimate. The certainty of evidence for the primary outcome was assessed using the Grading of Recommendations Assessment, Development and Evaluation (GRADE) framework. The evaluation considered five domains: risk of bias, inconsistency, indirectness, imprecision, and publication bias. The certainty of evidence was categorized as high, moderate, low, or very low. Detailed GRADE assessment results are presented in Supplementary File S4.

### 2.4. Statistical Analysis

Before conducting a meta-analysis, it is necessary to determine the effect size and test for heterogeneity in order to select an appropriate effect model. An effect size is a numerical value that quantifies the intensity of a phenomenon; the larger its absolute value, the stronger the effect and the more pronounced the phenomenon (Kelley & Preacher, 2012). When comparing differences in continuous outcomes between two groups, the standardized mean difference (SMD) is commonly used, and Cohen’s d is generally employed as the effect size. When a study includes small-sample studies (*n* < 60), Cohen’s d can significantly overestimate the effect size; therefore, the value must be adjusted. Multiplying the standardized mean difference (Cohen’s d) by a correction factor (J) yields an unbiased estimator (Hedges’ g),which is used as the effect size (Durlak, 2009).

After selecting the effect size, a heterogeneity test is conducted on the results of multiple studies to determine whether there is a genuine difference in the effect sizes. When studies exhibit high heterogeneity, a random-effects model is used to combine the effect sizes; when studies exhibit low heterogeneity, a fixed-effects model is used. Data processing for the meta-analysis was performed using Comprehensive Meta-Analysis (CMA) version 3.0 (Biostat, Inc., Englewood, NJ, USA).

## 3. Results

### 3.1. Search Results

**Figure 1:**
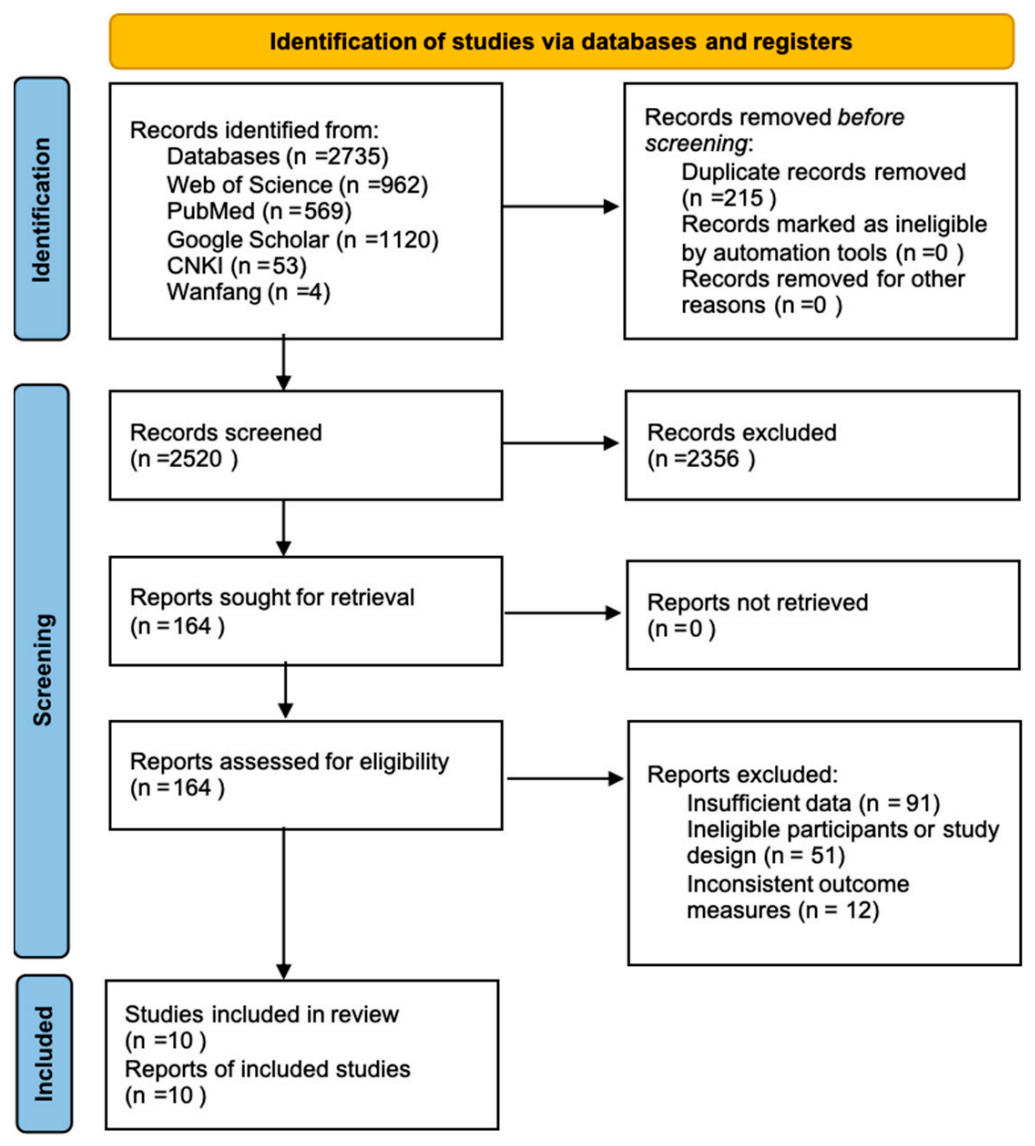
PRISMA 2020 flowchart of literature searches and study selection.

A total of 2735 articles were identified through a database search. After screening the articles based on the inclusion and exclusion criteria, 10 articles were ultimately included. The literature screening process and results are shown in Figure 1.

### 3.2. Characteristics of the Studies

The 10 included studies were published between 2017 and 2025 and compared people’s acceptance of conventional psychotherapy and AI-based psychotherapy. The sample size of the included articles ranged from 8 to 428, with a total of 1967 participants. The research subjects included college students, the elderly, and patients with mental disorders, etc. The conventional psychotherapy methods included human therapists’ treatment and bibliotherapy. The tools for using AI in psychotherapy included general conversational types (such as ChatGPT) and professional psychological types (such as Woebot). All included studies assessed acceptance-related outcomes using Likert-type scales, although the specific instruments and response ranges varied across studies. Because raw scores from different measurement tools are not directly comparable, standardized mean differences (Hedges’ g) were calculated to account for differences in measurement scales. Therefore, the pooled effect sizes represent standardized differences rather than direct comparisons of original questionnaire scores. Although some individual studies showed relatively large effect sizes, these estimates were derived from standardized mean differences (Hedges’ g), which adjust for differences in measurement scales and variability across studies. Therefore, the magnitude of these effects should be interpreted as standardized differences between AI-based psychotherapy and conventional psychotherapy groups rather than as a direct reflection of the original questionnaire score differences. The basic characteristics of the included literature are shown in Table 1.

### 3.3. Risk of Bias Assessment

The risk of bias assessment using the Kmet criteria indicated that all 10 included studies showed a generally low risk of bias. Detailed evaluations across individual domains and studies are presented in Supplementary File S3. As no study was classified as having a high risk of bias, risk-of-bias-based exclusion sensitivity analysis was not conducted.

### 3.4. Data Analysis

**Figure 2:**
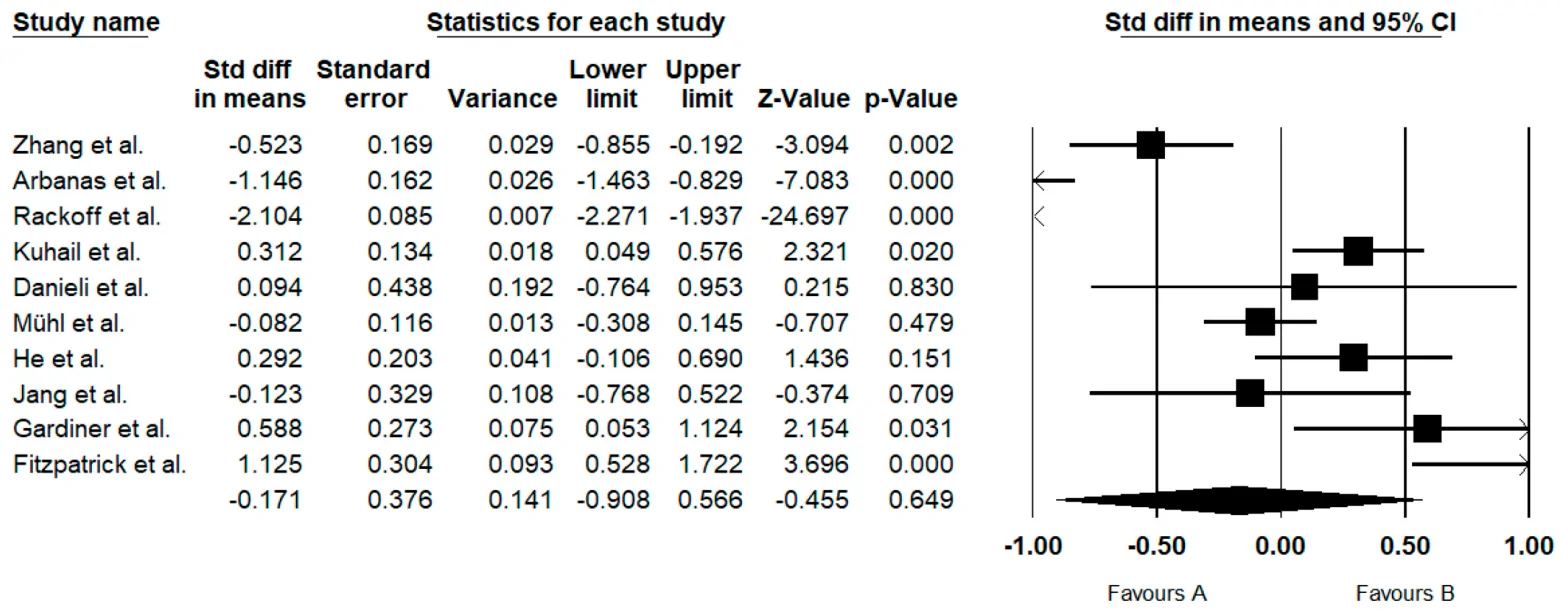
Forest plot comparing the acceptance of the AI group and the control group. Studies included Zhang et al.; Arbanas et al.; Rackoff et al.; Kuhail et al.; Danieli et al.; Mühl et al.; He et al.; Jang et al.; Gardiner et al.; Fitzpatrick et al. Note: “Favours A” indicates greater acceptance of conventional psychotherapy, whereas “Favours B” indicates greater acceptance of AI-based psychotherapy.

All 10 included studies reported on the acceptability of conventional psychotherapy and AI-assisted psychotherapy, involving a total of 1967 participants (AI group = 981, control group = 986). Heterogeneity tests revealed substantial heterogeneity among the studies (I^2^ = 98.0%, *p* < 0.001); therefore, a random-effects model was used to pool the effect sizes. The meta-analysis results showed that the overall effect size was g = −0.171 (95% CI: [−0.908, 0.566], *p* = 0.649), which did not reach statistical significance, as shown in Figure 2. Therefore, the pooled results did not provide sufficient evidence to conclude that AI-based psychotherapy has a clear advantage or disadvantage compared with conventional psychotherapy. Given the extremely high heterogeneity among studies, this finding should be interpreted with caution.

### 3.5. Sensitivity Analysis

**Figure 3:**
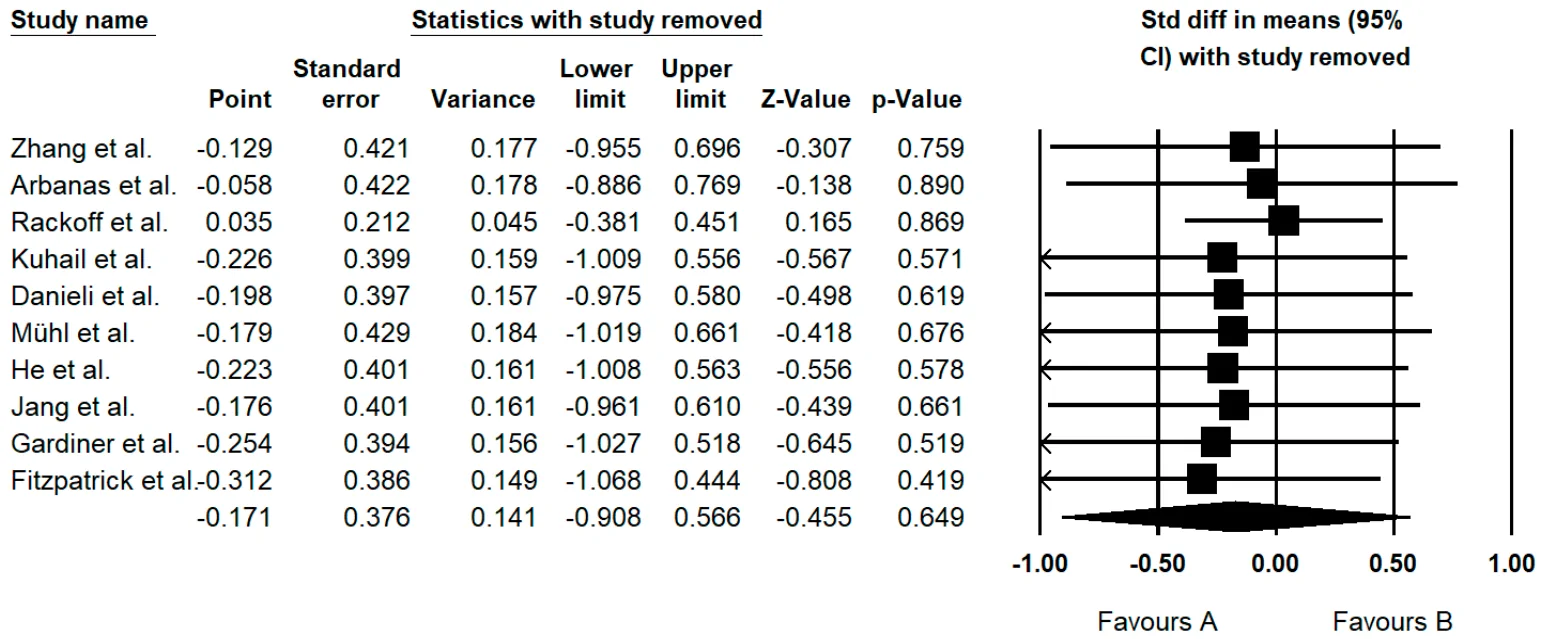
Leave-one-out sensitivity analysis of the pooled effect size. Studies included Zhang et al.; Arbanas et al.; Rackoff et al.; Kuhail et al.; Danieli et al.; Mühl et al.; He et al.; Jang et al.; Gardiner et al.; Fitzpatrick et al. Note: “Favours A” indicates greater acceptance of conventional psychotherapy, whereas “Favours B” indicates greater acceptance of AI-based psychotherapy.

A leave-one-out sensitivity analysis was conducted to examine whether the pooled effect size was disproportionately influenced by any individual study. The results showed that removing any single study did not substantially alter the overall effect estimate. The recalculated Hedges’ g values ranged from −0.312 to 0.035, and all effects remained statistically non-significant (all *p* > 0.05), suggesting that the pooled result was robust. The results are showed in Figure 3.

### 3.6. Heterogeneity Analysis

Due to the heterogeneity observed in the meta-analysis, it was necessary to further investigate the sources of this heterogeneity. Subgroup analyses were conducted for categorical variables such as control group therapy type, population, presence of psychological distress at baseline, participant source, cultural background, and AI type. Meta-regression analyses were performed for continuous variables such as mean age, year of publication, and sample size. The results showed that through subgroup analyses, the population, presence of psychological distress at baseline, and the source of participants could not explain the sources of heterogeneity, while control group therapy type, cultural background, and AI type may contribute to heterogeneity (*p* < 0.05). However, the cultural subgroup findings should be considered exploratory because some categories were represented by only one study. Meta-regression analysis indicated that mean age was not significantly associated with effect sizes, whereas publication year and sample size showed significant associations with effect sizes (*p* < 0.05). However, given the limited number of included studies, these meta-regression findings should be interpreted cautiously. The results are presented in Table 2 and Table 3.

### 3.7. Certainty of Evidence

The certainty of evidence for the primary outcome was assessed using the Grading of Recommendations Assessment, Development and Evaluation (GRADE) framework. The overall certainty of evidence for the acceptance of AI-based psychotherapy compared with conventional psychotherapy was rated as very low (⊕ ○ ○ ○). The certainty was downgraded due to substantial inconsistency across studies (I^2^ = 98%), indirectness related to variations in AI types, participant characteristics, cultural contexts, and measurement approaches, imprecision caused by the limited number of included studies and wide confidence interval, and potential publication bias. The detailed GRADE assessment is presented in Supplementary File S4.

### 3.8. Publication Bias

**Figure 4:**
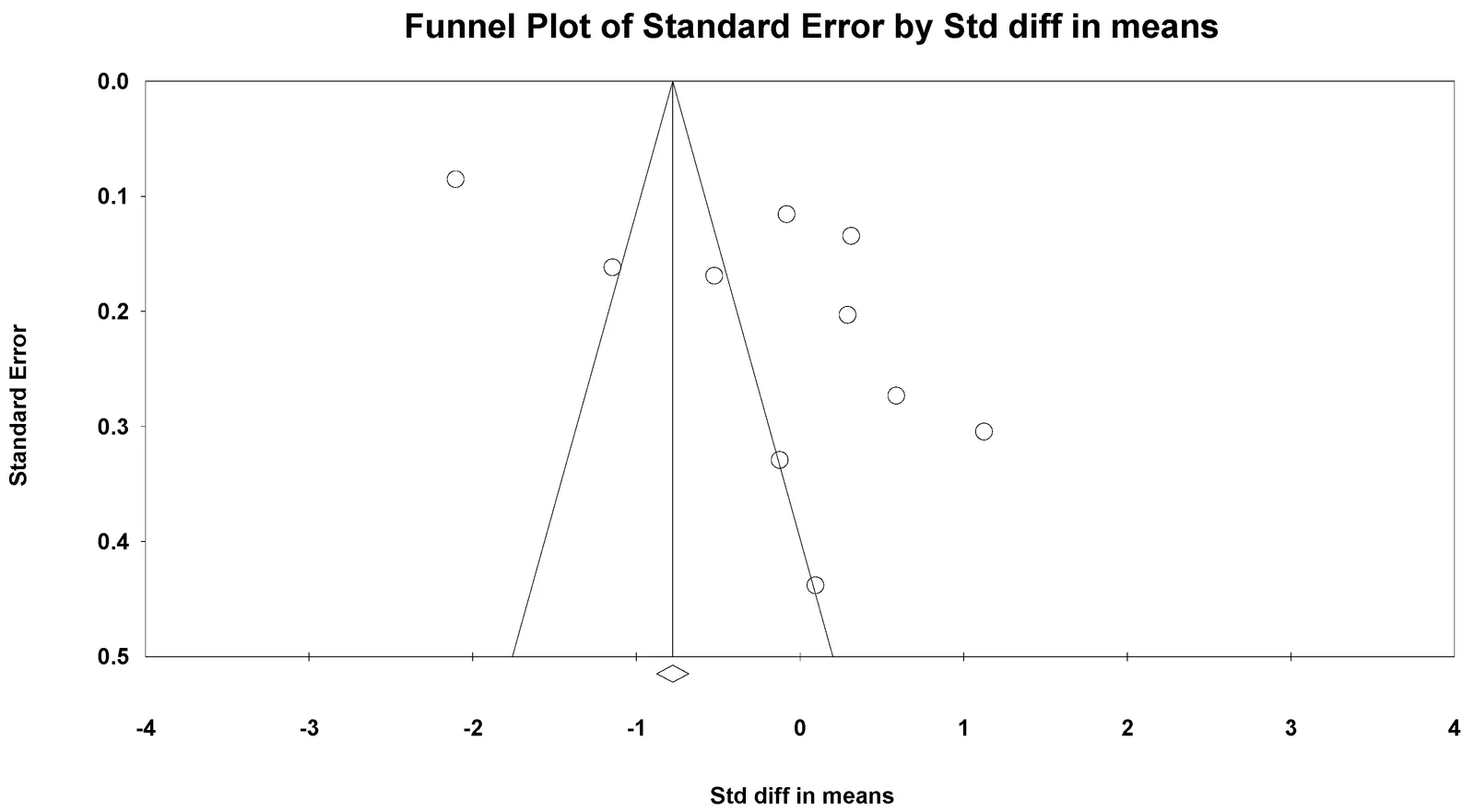
Funnel plot of the meta-analysis.

If the published literature does not have systematic representativeness, publication bias will occur (Rothstein et al., 2005), which will have a significant impact on the results of the meta-analysis. To address the issue of publication bias, a publication bias test was conducted. The distribution of the included studies in the funnel plot is shown in Figure 4. The funnel plot showed asymmetry, suggesting the possibility of publication bias. Egger’s regression test yielded a *p*-value of 0.055, which did not reach conventional statistical significance. However, given the limited number of included studies and the asymmetry observed in the funnel plot, publication bias cannot be ruled out.

## 4. Discussion

The results of this study indicate that the pooled effect did not demonstrate a statistically significant difference in acceptance between AI-assisted psychotherapy and conventional interventions. The analysis reveals that the absence of a significant difference in acceptance does not imply that the acceptance is homogeneous or stable. On the contrary, it was observed in the study that there is considerable heterogeneity (I^2^ = 98%). Through meta-analysis, potential sources were identified, mainly including the following aspects.

### 4.1. Type of Therapy in the Control Group

This study found that there was a significant difference in the therapy types between the control groups, indicating that the type of therapy used in the control group might be a factor influencing the heterogeneity of acceptance. There was no significant difference in acceptance of using AI for psychological treatment compared to human therapists; however, the acceptance of AI was higher than that of bibliotherapy. This may be because bibliotherapy interventions, as self-help methods, involve only psychoeducational concepts and do not provide a comprehensive psychotherapy program (Gregory et al., 2004), and the included studies did not design multiple or repeated sessions for participants (He et al., 2022), resulting in a lack of guidance. Consequently, when seeking psychotherapy, people may be more inclined to accept AI tools.

### 4.2. Cultural Background

This study found that there were significant differences in cultural backgrounds, suggesting that culture might be a factor influencing the heterogeneity of acceptance. In East Asian and Western cultures, there was no significant difference in the acceptance of AI-assisted psychotherapy compared to conventional psychotherapy; in the present analysis, the Southern European and Middle Eastern subgroups showed different patterns of acceptance. However, these findings should be interpreted cautiously because each subgroup was represented by only one study, and therefore they should be regarded as preliminary observations rather than evidence of stable cultural effects. The samples in the Southern European culture were from Croatia, Bosnia and Herzegovina, all of which were constituent units of the former Socialist Federal Republic of Yugoslavia. This federation disintegrated in the early 1990s, splitting into several sovereign states. Since 1995, people have been living in a post-conflict society undergoing economic transition. The value system has undergone significant changes, and people have to cope with immense and long-term traumas (Kucukalic et al., 2005). These countries have established comprehensive mental health systems centered around community mental health centers, where teams typically include psychiatrists, psychologists, nurses, and others, ensuring that mental health services reach the community (Jensen & Ceric, 1994; World Health Organization, 1996). One possible explanation is that established mental health care systems and familiarity with conventional services may influence preferences toward traditional psychotherapy. However, this interpretation remains speculative and requires further investigation.

However, some countries in the Arabian Peninsula are deeply conservative, and within Arab culture, there has traditionally been limited attention paid to mental health issues (Selim & Assiri, 2025), creating barriers to seeking mental health assistance. These barriers are closely linked to stigma, lack of awareness, confidentiality concerns, and negative attitudes stemming from cultural and religious beliefs (Alhumaidan et al., 2024). However, in recent years, the continuous development of AI technology has provided opportunities for mental health interventions through digital means. Due to the region’s unique geographical and historical context, gender inequality is relatively widespread (Mi, 2017), and attitudes toward the application of AI in mental health treatment differ between genders for distinct reasons. For men, their positive perception of AI as a learning tool is attributed to traditional gender roles and socialization patterns (Ofosu-Ampong, 2023); For women, who have long been in a state of “invisibility and silence,” facing significant oppression and being heavily constrained by traditional customs, technological advancements have created opportunities to reduce stigma and facilitate their access to mental health support (Aldaweesh, 2024). Therefore, the higher acceptance observed in the Middle Eastern subgroup may reflect characteristics of the specific study context rather than representing a general pattern across Middle Eastern cultures.

### 4.3. Types of AI Tools

This study found that there were significant differences in the types of AI tools, indicating that the type of AI tool might be a factor influencing the heterogeneity of acceptance. When the AI tools used by people were of the general conversation type, such as ChatGPT, they tended to prefer conventional psychotherapy; when the AI tools used were of the professional psychological type, they were more inclined to use AI for psychological treatment. This phenomenon may arise from a certain gap between the progress of psychology and artificial intelligence in the field of digital mental health (Lim et al., 2022). Due to the specialized nature of psychology, the expected outcomes of using general conversation-based AI cannot precisely assist users compared to conventional therapy, resulting in lower acceptance rates. In recent years, numerous mental health chatbots have emerged, offering interventions tailored to specific mental health issues. For example, Woebot targets depression and anxiety symptoms, Shim addresses stress and quality of life, and MYLO addresses general psychological distress (He et al., 2022), making them more targeted than conventional treatment methods. From the perspective of the Technology Acceptance Model, this disparity can be attributed to a systematic divergence in users’ perceived usefulness and perceived ease of use regarding the two types of tools (Davis, 1989). In terms of perceived usefulness, professional psychological AI tool conveys clear functional signals to users through explicit symptom interventions and professional positioning, significantly reducing cognitive effort and thereby enhancing perceived usefulness. Although general conversation AI tools are powerful, their non-specialized positioning makes it difficult for users to clearly perceive their usefulness. In the highly specialized context of psychological therapy, the outcomes users anticipate often fail to precisely align with their needs, resulting in lower acceptance rates. In terms of perceived ease of use, in mental health scenarios, the interface design of professional psychological AI imposes a lower cognitive load, whereas general conversational AI does the opposite. Taking Woebot and ChatGPT as examples, interactions with professional psychological AI are primarily driven by drop-down menus, topic cards, and binary choices. Users select from predefined topics within the interface without needing to formulate complex sentences, reducing the burden of description and resulting in higher perceived ease of use. In contrast, interactions with general conversational AI involve open-ended dialogue boxes that require users to describe their issues themselves, which may lead to difficulties in description within mental health contexts. Additionally, during psychotherapy, due to concerns about privacy and trust, many people prefer interacting with machines rather than humans, as they find it easier to disclose personal information to a machine (Aktan et al., 2022; Topol, 2019). Provided that professional standards are maintained, people may be more willing to accept AI-based psychotherapy. However, differences in acceptance may not be fully explained by the functional characteristics of AI systems alone. Users’ acceptance may also depend on how they cognitively interpret and socially understand these technologies.

### 4.4. Technological Cognition and Human–AI Interaction

Beyond perceived usefulness and ease of use, differences in acceptance across AI systems may also reflect variations in how users cognitively represent digital technologies. Recent perspectives on technological cognition suggest that humans do not process all technologies in the same manner. Unlike mechanical tools, which are primarily understood through action-related knowledge and sensorimotor experience, digital technologies require users to rely more heavily on conceptual understanding and social cognition when interacting with them (Federico et al., 2025).

This distinction may be particularly relevant for AI-based psychotherapy. Unlike conventional digital tools that mainly provide functional assistance, AI psychotherapy systems engage users through conversational interaction and may therefore be perceived not only as technological instruments but also as interactive social entities. Users may evaluate such systems based on whether they appear understandable, trustworthy, emotionally responsive, and capable of providing meaningful support.

The integrated framework of technological cognition further proposes that human–technology interaction is supported by multiple cognitive processes, including causal reasoning, semantic cognition, sensorimotor knowledge, and social learning (Federico et al., 2026). This perspective suggests that acceptance of AI-based psychotherapy may not depend solely on the functional performance of AI systems, but also on how users cognitively interpret the role and meaning of AI within therapeutic contexts. Specifically, users may differ in whether they perceive AI as merely a digital tool, an informational assistant, or an interactive therapeutic partner, which may subsequently influence trust, perceived agency, and willingness to engage with AI-based psychotherapy. From this perspective, acceptance of AI psychotherapy may depend not only on the objective capabilities of AI systems but also on users’ mental representations of what AI is and what role it can play in psychological support.

Therefore, the substantial heterogeneity observed in this meta-analysis may partly arise from differences in users’ technological cognition and their interpretations of AI’s role in psychotherapy. General conversational AI systems, such as ChatGPT, may be interpreted as broad-purpose digital assistants rather than professional psychological agents, whereas specialized mental health chatbots may provide clearer therapeutic identities and expectations. This cognitive distinction may contribute to variations in perceived usefulness, trust, and willingness to engage with AI-based psychotherapy.

### 4.5. The Year of Publication

The meta-regression analysis showed a significant negative association between publication year and effect size, with later studies reporting relatively lower acceptance of AI-based psychotherapy. However, given the limited number of studies included in the meta-regression, this association should be regarded as exploratory and interpreted cautiously. This may be attributed to the influence of perceived novelty in the early stages of AI adoption: perceived novelty is a factor that positively influences the willingness to adopt information, reflecting users’ willingness to accept new things and their individual perceptions (Huo & Zhao, 2025). Consequently, public acceptance of AI has shifted from an initial sense of novelty to a later stage of rational evaluation. This shift may also be attributed to the fact that later studies featured more rigorous designs and more representative samples.

### 4.6. Sample Size

The meta-regression analysis also showed a significant negative association between sample size and effect size, with larger studies reporting relatively lower acceptance of AI-assisted psychotherapy. Given the limited number of studies available for meta-regression, this finding should be interpreted cautiously. The observed findings suggest small-study bias, that is, small-study effects, rather than sample size per se, may account for the variations in acceptance outcomes. When a meta-analysis includes a large number of “small” trials, it may be misleading because the estimated intervention effects in these trials are likely to be overestimated (Cappelleri et al., 1996; Dechartres et al., 2013; Ioannidis et al., 1998). That is, studies with smaller sample sizes and relatively lower methodological rigor may overestimate effect sizes, thereby leading to publication bias. Furthermore, analysis of publication bias revealed a distinctly asymmetrical funnel plot, suggesting the presence of publication bias. Although Egger’s regression test did not reach statistical significance (*p* = 0.055), the borderline *p*-value combined with the asymmetry observed in the funnel plot suggests a potential risk of publication bias. Therefore, publication bias cannot be ruled out, and the findings should be interpreted cautiously.

This study has certain limitations. First, the meta-analysis only included 10 studies, which limits the statistical power of several analyses. Specifically, the small number of included studies may reduce the reliability of publication bias assessments; therefore, the possibility of publication bias should be interpreted cautiously. The limited number of studies also restricted the statistical power of the meta-regression analyses, particularly because three continuous study-level covariates—mean age, publication year, and sample size—were examined. Accordingly, the associations identified in the meta-regression should be considered exploratory and hypothesis-generating rather than conclusive evidence of moderator effects. Furthermore, there were substantial differences in study size, with sample sizes ranging from 8 to 428 participants. The presence of numerous small-sample studies increases the risk of overestimating effect sizes, posing challenges to the robustness and generalizability of the conclusions. Nevertheless, the leave-one-out sensitivity analysis indicated that the pooled estimate was not substantially affected by the exclusion of any individual study, supporting the robustness of the overall findings. Second, all included studies assessed acceptance levels through questionnaire surveys, but the original texts rarely reported the reliability of the questionnaires, making it impossible to determine whether the individual items within the questionnaires were stable. In addition, the included studies used heterogeneous operationalizations of acceptance, ranging from behavioral intention and attitudes to satisfaction and trust-related evaluations. Although these indicators were considered conceptually related, differences in measurement approaches may have contributed to the observed heterogeneity. Furthermore, the studies spanned the years 2017 to 2025. Given the rapid development of artificial intelligence technology in recent years, older-generation models have limitations, and research results from several years ago may no longer reflect current capabilities (Föyen et al., 2025). Additionally, this review was not prospectively registered in a public registry, which may reduce the transparency of the review process. Future systematic reviews should consider prospective registration to further enhance methodological transparency. Finally, the study participants came from different countries, and differences in population characteristics cannot be ruled out; this factor may influence people’s acceptance of psychotherapy.

## 5. Conclusions

This meta-analysis synthesized 10 studies examining differences in people’s acceptance of AI-based versus conventional psychotherapy. Overall, the pooled analysis did not identify a statistically significant difference between AI-based psychotherapy and conventional psychotherapy. However, due to substantial heterogeneity across studies, this finding should not be interpreted as evidence that the two approaches are universally equivalent. Individual outcomes are influenced by multiple factors, including the type of conventional psychotherapy, cultural background, the type of AI, user perceptions, and even the maturity of the technology.

The phenomenon of differing acceptance is complex: it involves users balancing professionalism against privacy. When comparing AI therapy to human therapists, it cannot yet be simply concluded that one is superior to the other. Human therapists have their own unique value, particularly in providing warm, empathetic responses that offer a genuine sense of being heard and validated. Meanwhile, the integration of AI technology can serve as a complementary tool, helping individuals overcome barriers such as time constraints, cost, or feelings of shame. It can facilitate smoother self-disclosure to machines and even reduce the sense of shame associated with interpersonal interactions, demonstrating potential advantages over conventional mental health services.

Furthermore, cultural context may represent one potential factor associated with variations in acceptance; however, its role should be further examined using larger samples and more culturally diverse studies. The acceptance of AI-assisted psychotherapy depends not only on the maturity of the technology but also on its application within a specific cultural context. The integration of AI technology can serve as a privacy-preserving outlet that breaks free from traditional constraints; conversely, conventional therapeutic approaches can represent a continuation of interpersonal trust in the process of social reconstruction. This cultural heterogeneity suggests that future promotion of AI technology—particularly in the field of psychotherapy—must take cultural sensitivity into account, integrating the social significance and emotional nuances underlying mental health issues to avoid cold, hasty judgments and the imposition of a one-size-fits-all model.

In summary, discussions regarding the acceptance of AI-assisted psychotherapy should avoid sweeping generalizations and must fully account for the influence of multidimensional factors. Future research requires larger sample sizes and methodologically rigorous studies to provide more robust and less biased evidence.

## Figures and Tables

**Table 1:** Basic Characteristics of Included Studies.

Study ID	Year	Region	Conventional Psychotherapy				Psychotherapy Using AI					
			Therapy Method	*n*	Mean	SD	Type of AI	*n*	Mean	SD	Acceptance-Related Outcome	Measurement Instrument
(Zhang & Lian, 2025)	2025	China	human therapist	76	5.58	1.08	general conversational type	69	5.01	1.10	Intention	Consultation intention scale (7-point Likert scale)
(Arbanas et al., 2025)	2025	Croatia, Bosnia and Herzegovina	human therapist	89	4.67	0.64	general conversational type	89	3.66	1.07	Satisfaction	Study-specific questionnaire (5-point Likert scale)
(Rackoff et al., 2025)	2025	the U.S.	human therapist	428	5.78	1.08	general conversational type	428	3.40	1.18	Attitudes	Mental Help Seeking Attitudes Scale (MHSAS, 7-point Likert scale)
(Kuhail et al., 2024)	2024	Saudi Arabia	human therapist	112	3.09	1.15	professional psychological type	112	3.46	1.22	Satisfaction	Study-specific questionnaire (5-point Likert scale)
(Danieli et al., 2022)	2022	Italy	human therapist	15	4.21	0.89	professional psychological type	8	4.29	0.76	Satisfaction	Study-specific questionnaire (5-point Likert scale)
(Mühl et al., 2024)	2024	Germany	human therapist	145	3.57	0.30	professional psychological type	155	3.54	0.42	Trust	Social Trust in Therapy (SV-TAB) subscale of the Short Scales to Assess Trust in Psychotherapy (VTT-TABs) (5-point Likert scale)
(He et al., 2022)	2022	China	bibliotherapy	49	4.43	0.89	professional psychological type	49	4.67	0.75	Acceptability	Acceptability Scale (AS, 5-point Likert scale)
(Jang et al., 2021)	2021	South Korea	bibliotherapy	18	2.17	0.85	professional psychological type	19	2.05	1.08	Acceptability	Study-specific questionnaire (5-point Likert scale)
(Gardiner et al., 2017)	2017	the U.S.	bibliotherapy	29	5.00	1.60	professional psychological type	27	6.00	1.80	Satisfaction	Study-specific questionnaire (7-point Likert scale)
(Fitzpatrick et al., 2017)	2017	the U.S.	bibliotherapy	25	3.40	0.80	professional psychological type	25	4.30	0.80	Acceptability	Study-specific questionnaire (5-point Likert scale)

Note: SD = standard deviation; AI = artificial intelligence.

**Table 2:** Results of Subgroup Analysis.

Variable	*N*	Effect Size	Two-Tailed Test		95% CI		Between-Group *p*-Value
			*Z*-Value	*p*-Value	Lower	Upper	
the type of therapy in the control group							
human therapist	6	−0.589	−1.245	0.213	−1.517	0.338	*p* = 0.045 *
bibliotherapy	4	0.470	2.010	0.044	0.012	0.928	
population							
college students	5	−0.192	−0.295	0.768	−1.464	1.081	*p* = 0.312
clinical patients	2	−0.675	−1.323	0.186	−1.674	0.324	
general population	3	0.165	0.701	0.483	−0.297	0.628	
Baseline Psychological Distress							
yes	7	−0.198	−0.375	0.707	−1.233	0.836	*p* = 0.847
no	3	−0.088	−0.399	0.690	−0.518	0.343	
Participant Source							
clinical sample	3	−0.243	−0.420	0.674	−1.377	0.891	*p* = 0.892
community sample	7	−0.140	−0.288	0.773	−1.093	0.813	
Cultural Background							
East Asian Culture	3	−0.126	−0.447	0.655	−0.681	0.428	*p* < 0.001 ***
Southern European Culture	1	−1.146	−7.083	<0.001	−1.463	−0.829	
Western Culture	5	−0.091	−0.134	0.893	−1.424	1.241	
Middle Eastern Culture	1	0.312	2.321	0.020	0.049	0.576	
AI Type							
general conversational type	3	−1.265	−2.508	0.012	−2.254	−0.277	*p* = 0.003 **
professional psychological type	7	0.295	2.005	0.045	0.007	0.584	

Note: * *p* < 0.05, ** *p* < 0.01; *** *p* < 0.001.

**Table 3:** Results of Meta-regression Analysis.

Variable	Coefficient	Standard Error	95%CI	*p*-Value
Mean Age	0.0028	0.0364	−0.0686, 0.0742	0.9391
Publication Year	−0.2234	0.1045	−0.4283, −0.0186	0.0325
Sample Size	−0.0057	0.0017	−0.0090, −0.0025	0.0006

## Data Availability

All analyzed data in this meta-analysis were derived from published peer-reviewed studies. No new original data were generated in this work.

## Supplementary Materials

The following supporting information can be downloaded at: https://www.mdpi.com/article/10.3390/bs16091552/s1, File S1: PRISMA 2020 checklist; File S2: Full Search Strategies; File S3: Risk of bias assessment for the included studies; File S4: Summary of findings and certainty of evidence (GRADE assessment). Reference (Page et al., 2021) is cited in the supplementary materials.
