# Supplementary material for: A Meta-Analysis of the Acceptance of Artificial Intelligence in Psychotherapy
Source: Behav Sci (Basel). 2026 Sep 2;16(9):1552. doi: 10.3390/bs16091552 (PMC13603263; doi:10.3390/bs16091552)
Supplement: Supplementary file 1 [file behavsci-16-01552-s001.zip › Supplementary S2. Full Search Strategies.pdf]

Web of Science

("AI" OR "artificial intelligence" OR "chatbot" OR "conversational agent")
AND
("mental health" OR "psychotherapy" OR "counseling")
AND
("attitude" OR "acceptance" OR "acceptability" OR "satisfaction")

PubMed

("AI" OR "artificial intelligence" OR "chatbot" OR "conversational agent" OR "ChatGPT" OR "large language model" OR "LLM" OR "generative AI")
AND
("mental health" OR "psychotherapy" OR "counseling")
AND
("attitude" OR "acceptance" OR "acceptability" OR "satisfaction")

Google Scholar

("AI" OR "artificial intelligence" OR chatbot OR "conversational agent" OR ChatGPT OR "large language model" OR LLM OR "generative AI")
AND
("mental health" OR psychotherapy OR counseling)
AND
(attitude OR acceptance OR acceptability OR satisfaction)

CNKI

("人工智能" OR AI OR ChatGPT OR "大语言模型" OR LLM OR "生成式人工智能" OR "聊天机器人")
AND
("心理治疗" OR "心理咨询" OR "精神健康")

AND

("接受度" OR "接受意愿" OR "态度" OR "可接受性" OR "满意度")

Wanfang

("人工智能" OR AI OR ChatGPT OR "大语言模型" OR LLM OR "生成式人工智能" OR "聊天机器人")

AND

("心理治疗" OR "心理咨询" OR "精神健康")

AND

("接受度" OR "接受意愿" OR "态度" OR "可接受性" OR "满意度")
