# Supplementary material for: A Meta-Analysis of the Acceptance of Artificial Intelligence in Psychotherapy
Source: Behav Sci (Basel). 2026 Sep 2;16(9):1552. doi: 10.3390/bs16091552 (PMC13603263; doi:10.3390/bs16091552)
Supplement: Supplementary file 1 [file behavsci-16-01552-s001.zip › Supplementary S3. Risk of bias assessment for the included studies..pdf]

Risk of bias assessment for the included studies.

Scoring criteria: 2 = Yes; 1 = Partial; 0 = No; NA = Not applicable.

Study	Q1	Q2	Q3	Q4	Q5	Q6	Q7	Q8	Q9	Q10	Q11	Q12	Q13	Q14	Total
Zhang and Lian, 2025	2	2	1	2	2	1	1	2	1	2	2	2	2	2	24
Arbanas et al., 2025	2	2	1	2	NA	NA	NA	2	1	2	2	1	2	2	19
Rackoff et al., 2025	2	2	1	2	NA	NA	NA	2	1	2	2	1	2	2	19
Kuhail et al., 2024	2	2	1	2	2	1	2	2	1	2	2	1	2	2	24
Danieli et al., 2022	2	2	1	2	2	0	0	2	1	2	2	1	2	2	21
Mühl et al., 2024	2	2	2	2	2	1	1	2	2	2	2	2	2	2	26
He et al., 2022	2	2	2	2	2	2	2	2	2	2	2	2	2	2	28
Jang et al., 2021	2	2	2	2	2	0	0	2	1	2	2	1	2	2	22
Gardiner et al., 2017	2	2	2	2	2	0	0	2	1	2	2	1	2	2	22
Fitzpatrick et al., 2017	2	2	2	2	2	1	0	2	2	2	2	2	2	2	25

Q1: Question/objective sufficiently described？

Q2: Study design evident and appropriate？

Q3: Method of subject/comparison group selection or source of information/input variables described and appropriate？

Q4: Subject (and comparison group, if applicable) characteristics sufficiently described？

Q5: If interventional and random allocation was possible, was it described？

Q6: If interventional and blinding of investigators was possible, was it reported？

Q7: If interventional and blinding of subjects was possible, was it reported？

Q8: Outcome and (if applicable) exposure measure(s) well defined and robust to measurement /misclassification bias? Means of assessment reported？

Q9: Sample size appropriate？

Q10: Analytic methods described/justified and appropriate?

Q11: Some estimate of variance is reported for the main results？

Q12: Controlled for confounding?

Q13: Results reported in sufficient detail？

Q14: Conclusions supported by the results?
