# Supplementary material for: A Meta-Analysis of the Acceptance of Artificial Intelligence in Psychotherapy
Source: Behav Sci (Basel). 2026 Sep 2;16(9):1552. doi: 10.3390/bs16091552 (PMC13603263; doi:10.3390/bs16091552)
Supplement: Supplementary file 1 [file behavsci-16-01552-s001.zip › Supplementary S4.pdf]

Summary of findings and certainty of evidence (GRADE assessment)

CI = confidence interval; GRADE = Grading of Recommendations Assessment, Development and Evaluation. Certainty of evidence is expressed using

Outcome	Studies and Participants	Pooled Effect Estimate	Certainty of Evidence
acceptance of AI-based psychotherapy compared with conventional psychotherapy	10 studies; n = 1967	Hedges' g = −0.171 (95% CI: −0.908 to 0.566)	Very low⊕ ○ ○ ○ [a]

GRADE symbols, where ⊕ ⊕ ⊕ ⊕ indicates high certainty, ⊕ ⊕ ⊕ ○ indicates moderate certainty, ⊕ ⊕ ○ ○ indicates low certainty, and ⊕ ○ ○ ○ indicates very low certainty.

[a] Downgraded for very serious inconsistency due to substantial heterogeneity (I^2 = 98%), serious indirectness due to variations in AI systems, populations, cultural contexts, and measurement approaches; serious imprecision due to limited number of studies; and suspected publication bias.
